# Mobile learning for HIV/AIDS healthcare worker training in resource-limited settings

**DOI:** 10.1186/1742-6405-7-35

**Published:** 2010-09-08

**Authors:** Maria Zolfo, David Iglesias, Carlos Kiyan, Juan Echevarria, Luis Fucay, Ellar Llacsahuanga, Inge de Waard, Victor Suàrez, Walter Castillo Llaque, Lutgarde Lynen

**Affiliations:** 1 Institute of Tropical Medicine, Antwerp, Belgium; 2 Institute of Tropical Medicine Alexander von Humboldt, Lima, Peru; 3 National Institute of Health, Lima, Peru

## Abstract

**Background:**

We present an innovative approach to healthcare worker (HCW) training using mobile phones as a personal learning environment.

Twenty physicians used individual Smartphones (Nokia N95 and iPhone), each equipped with a portable solar charger. Doctors worked in urban and peri-urban HIV/AIDS clinics in Peru, where almost 70% of the nation's HIV patients in need are on treatment. A set of 3D learning scenarios simulating interactive clinical cases was developed and adapted to the Smartphones for a continuing medical education program lasting 3 months. A mobile educational platform supporting learning events tracked participant learning progress. A discussion forum accessible via mobile connected participants to a group of HIV specialists available for back-up of the medical information. Learning outcomes were verified through mobile quizzes using multiple choice questions at the end of each module.

**Methods:**

In December 2009, a mid-term evaluation was conducted, targeting both technical feasibility and user satisfaction. It also highlighted user perception of the program and the technical challenges encountered using mobile devices for lifelong learning.

**Results:**

With a response rate of 90% (18/20 questionnaires returned), the overall satisfaction of using mobile tools was generally greater for the iPhone. Access to Skype and Facebook, screen/keyboard size, and image quality were cited as more troublesome for the Nokia N95 compared to the iPhone.

**Conclusions:**

Training, supervision and clinical mentoring of health workers are the cornerstone of the scaling up process of HIV/AIDS care in resource-limited settings (RLSs). Educational modules on mobile phones can give flexibility to HCWs for accessing learning content anywhere. However lack of softwares interoperability and the high investment cost for the Smartphones' purchase could represent a limitation to the wide spread use of such kind mLearning programs in RLSs.

## Background

"Mobile learning" or "mLearning" is learning that occurs across locations, benefiting of the opportunities that portable technologies offer. The term is most commonly used in reference to using PDAs, MP3 players, notebooks and mobile phones for health education and knowledge sharing. One definition of mobile learning is: *Any sort of learning that happens when the learner is not at a fixed, predetermined location, or learning that happens when the learner takes advantage of the learning opportunities offered by mobile technologies *[1] but another definition might be *learning in motion*. One issue that became clear is that mobile learning is not just about learning using portable devices, but *learning across contexts*, within diverse target groups, according to different learning design, development and implementation [2].

Healthcare workers (HCWs) have indicated the need for an autonomous mobile solution that would enable access to the latest medical information for continuing professional development using low-cost devices and facilitate exchange of ideas about difficult clinical cases with peers through social media [2,3]. As the most important social technology used worldwide, mobile devices in particular play a major role in stimulating this information exchange, and the advent of mobile and wireless technology has changed the level of information and communication technology (ICT) penetration in the resource-limited setting (RLSs) [4-7].

Peru does not have an adequate health care workforce to meet the population's demand for services and for the management and development of new human resources.

Limited development of health personnel competencies, health personnel in remote areas who lack access to training opportunities, poor coordination with training institutions whose training does not meet regional needs, training programs carried out in settings different from the actual work context, no performance evaluation based on competencies, high turnover rates for trained staff are major challenges identifies by national, regional, and local governments for the healthcare human resource development in Peru [8]. At the present the vast majority of health care professionals are operating in isolation from vital health information [9]. Access to reliable health information has been described as one of the most effective strategies for sustainable improvement in health care [10,11]. In this context, the Peruvian Ministry of Health (MOH) approved the Policy Guidelines on Human Resources in Health, which include tailoring training to the needs of the country, building competencies, decentralizing the management of human resources, and generating motivation and commitment. The training of service providers in all areas of HIV prevention, treatment and care is a significant component of the MOH programme to develop human potential [12].

The goal of this mLearning project was to enable HCWs involved in HIV/AIDS care in urban and peri-urban stations in Peru to access the state-of-the-art in HIV treatment and care. To achieve this aim, in 2008 the Institute of Tropical Medicine Alexander von Humboldt (IMTAvH) in Lima and the Institute of Tropical Medicine (ITM) in Antwerp set up an educational mobile application, allowing knowledge sharing and data contribution through a mobile-based educational platform.

## Materials and methods

**Figure 1:**
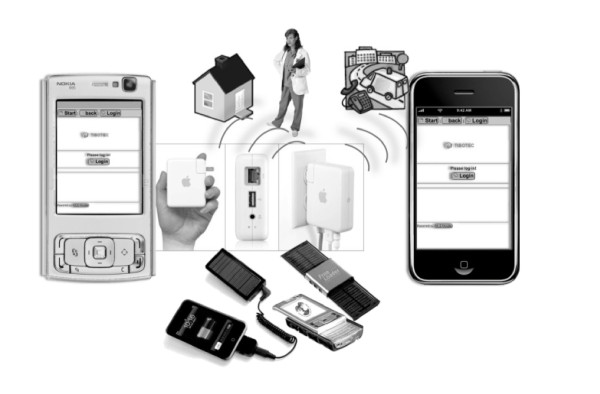
**Smartphones: Nokia N95 and iPhone**.

Of 24 Peruvian department capitals, 20 were already involved with the IMTAvH in a distance-learning project begun in 2004 and lasting a year with the aim to scale up access to antiretroviral treatment in the Peruvian peripheral regions. Some of these facilities were included in the mLearning pilot project. Health centers in the department capitals are run by medical doctors and staffed with 5-10 HCWs, such as social workers, counselors, and data clerks. Individual Smartphones (10 iPhones, mobile phone with touch-screen and 10 Nokia N95, mobile phone with digit buttons to dial with), each equipped with a portable solar charger, were delivered to the 20 physicians based in the peri-urban HIV centers. A router connected to a DSL or cable modem, available in all stations, allowed wireless connection, facilitating surfing and the downloading of the didactic material in any area of the clinic. This access also simultaneously guaranteed wire-free interactions, without participants having to purchase a complete computer to connect, and reducing the cost of communications by using Skype via mobiles (Figure 1).

The training program consisted of a set of "clinical modules" simulating interactive clinical cases that were adapted to mobile devices and sent to physicians working in the 20 peri-urban clinical stations. The case series involved five topic areas, the most common being the use of new drugs for HIV/AIDS treatment and their safety and side-effect profiles (see Additional file 1). The mLearning program was delivered during the months of November 2009-January 2010. Half-day training on how to operate with the mobile equipment was taken at IMTAvH by all participants before the launching of the mLearning program.

**Figure 2:**
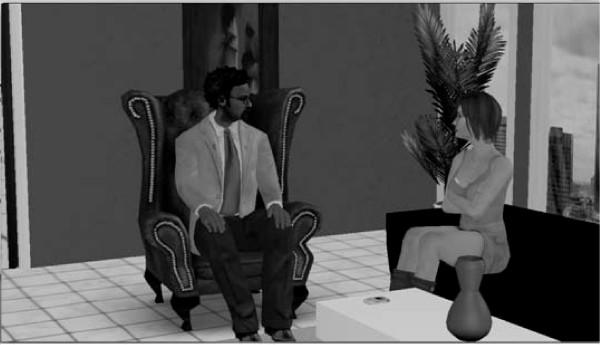
**Example of 3D animation**.

The didactic material used in this project was developed with 3D animations using iClone [13] and Moviestorm [14], reproducing specific scenarios (e.g., clinical consultation) (Figure 2) while the module revision at end of every case discussion was provided through multimedia files (developed with ScreenFlow [15], which enables starting from PowerPoint presentations to add audio and video to screen shots, and to publish everything in a mobile-accessible format).

**Figure 3:**
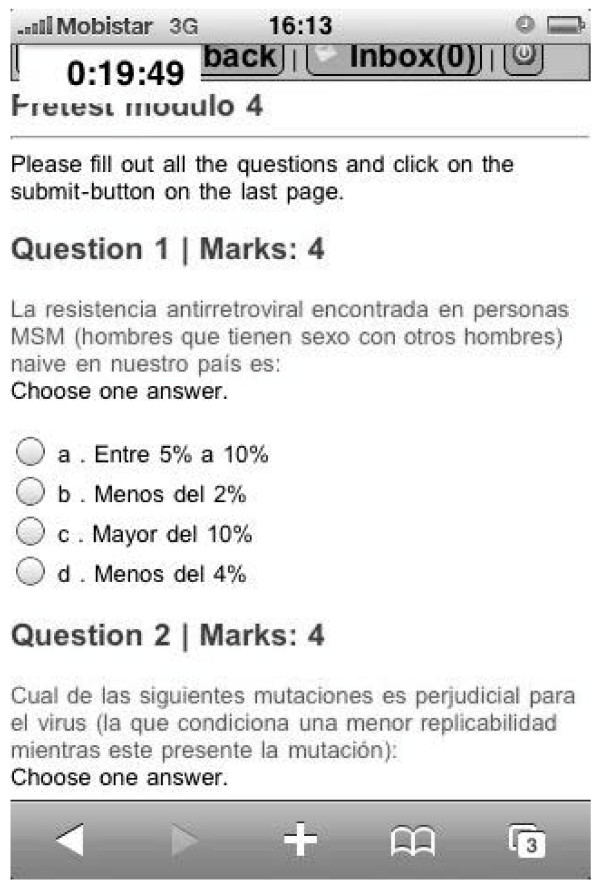
**Pre-test, example**.

Learning outcomes of the acquired knowledge were tested through mobile-based multiple choice questions (pre- and post-test) issued at the beginning and end of each module (Figure 3).

**Figure 4:**
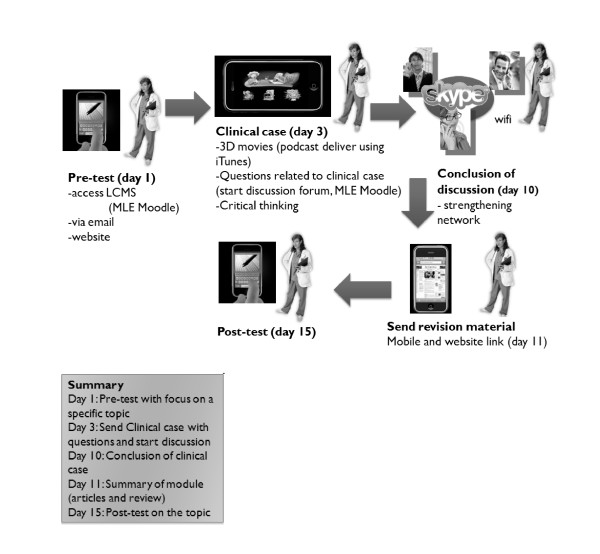
**Flow of the 5 clinical modules**.

A functional mobile platform (MLE Moodle) was offered to support the learning events, tracking student progress over time. The platform also provided access to Facebook for peer-to-peer learning sharing in clinical case discussions with a network of experts, which assured feedback content quality. The suggested readings were distributed within the timeframe of the 2-week clinical module discussion mainly in PDF format using Google Docs (Figure 4).

In December 2009, a mid-term user satisfaction survey delivered through a standardized anonymous questionnaire, coupled with a focus group discussion, was performed. The satisfaction survey sought to gain feedback on tutorial quality, usefulness of the information, and its applicability to the daily context of HIV treatment and care. The focus group discussion sought to identify general barriers to program adherence and the technical difficulties encountered during the implementation phase of the program.

## Results

**Figure 5:**
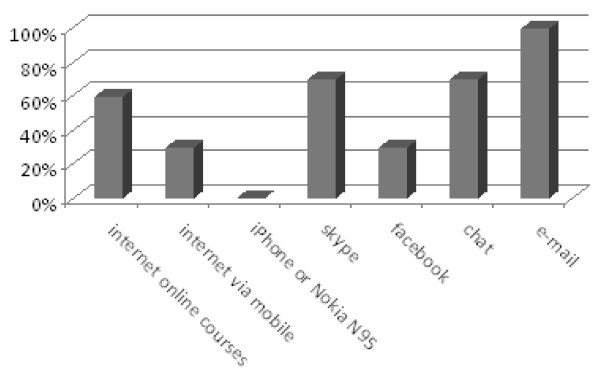
**Previous computer use among participants**.

Of the 20 participants, 18 returned the standardized questionnaires (response rate, 90%). Participant median age was 48.5 years (range, 34-55 years), with a median of 6 years of experience treating HIV patients. Most participants had no prior mobile learning experience, and their social media literacy was also limited (Figure 5).

**Figure 6:**
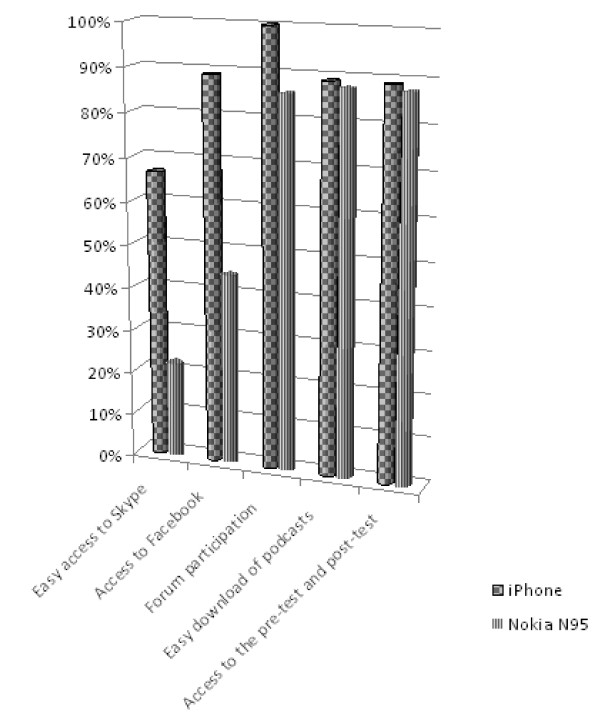
**Use of applications according to mobile device**.

Over half of the iPhone users (66.7%) indicated that Skype was easy to access compared to 22.2% using the Nokia N95; in addition, 88.9% of the iPhone respondents found it easy to access Facebook via mobile compared to the 44.4% using the Nokia N95. The results indicated similar usability of iPhone and Nokia N95 (88.9% and 87.5% respectively) for the download of podcasts and access to MLE Moodle for pre- and post-testing (Figure 6).

The freedom to plan educational activities according to each individual user's personal agenda was indicated as an added value by 86.6% of the participants, while 94.4% indicated that access to the educational content without needing a computer was an added value. All respondents had positive opinions about the quality of the received information, the applicability of the content to clinical practice, and the appropriate relevance of the suggested readings.

The main advantages participants identified during the focus group discussion were the portability of the equipment and easy access to the educational content at the time and location of their choice. Some of the Nokia N95 users reported as problematic the screen size of the equipment, the keyboard size, and the quality of the images. The topics covered by the program were graded as pertinent to daily clinical practice and highly regarded by the participants.

## Discussion

Many developing countries would move towards the use of distance-learning programs to avoid leaving peripheral health stations unstaffed when HCWs are absent for short or long training programs [16,17]. Because Peru is a developing country, there is limited access to information and teaching resources and a great need to enhance learning and teaching environments. Mobile phones can create an inexpensive and reliable learning environment between HCWs in one-to-one personal learning and between colleagues in a network [18]. Some of the mobile devices are relatively low cost, powerful, small, and lightweight, and they can perform well in difficult environments because of the limited power required by the battery, which can be recharged using inexpensive solar panels.

HCWs can learn to use mobile devices, search for information, and upload and download information in a relatively short time frame [19-21]. Smartphones enable users to upload and download information using a wireless network. The Smartphone can be very useful in distance learning, giving users the opportunity to contact a mentor by phone, receiving immediate feedback and helping to establish a network. This study showed the value of the use of mobile phones for personal education in RLSs. In addition, it attempted to compare performance of two different devices (touch-screen versus digit buttons) looking at screen and keyboard size and interoperability of the software applications of two different operating systems.

There was not a single mobile application able to provide all the different learning activities for both mobile devices, so different applications had to be used (e.g., MLE Moodle to provide pre- and post-test and Facebook for the discussion forum, Google Docs for document delivery).

After the pre-test on a specific subject the participants were challenged with a clinical case mirroring a real clinical situation developed in 3D (Figure 2). According to the learning objectives of every module the participants had to discuss some questions related to the topic using the Facebook discussion forum or Skype for a call. The most important points discussed were noted down and a final movie summarizing the most relevant information could be generated and made available together with the recommended readings links on the mobile phones. A post-test has been taken at the end of every module using MLE Moodle.

The overall satisfaction of using iPhone or Nokia N95 as expressed by the participants was generally greater for iPhone: the Nokia N95 users described access to Skype and Facebook as being more complicated, also expressing less satisfaction with the screen and the keyboard size and the quality of the images on this equipment.

The unique feature of this project is that technology was used bridging the gap between formal and experiential learning.

Three limitations need to be acknowledged and addressed. The first concerns the relatively high investment cost for purchasing the mobile devices, the phone service fee, and the need for an IT help desk to solve technical problems. The second limitation involves a lack of measure of the extent to which these findings can be generalized beyond the pilot project and the interoperability of those educational modules using other more basic phones.

This pilot project is a single case and we do not attempt to make a generalization of our results. More research is needed to understand if what observed can be applied to other mLearning programs moreover in RLSs. Our next step in this research will be to develop a survey with data triangulation using in depth interviews, group discussion and participants validation.

## Conclusions

Educational modules available via mobile computing give flexibility to the healthcare workers who can carry and access content anywhere. Mobile devices enhance the learning environment and strengthen the ability to share knowledge through online discussion via social media or directly by phone. The sharing of experiences in a network facilitates the transformation of learning outcomes into permanent and valuable knowledge assets.

These preliminary results show that the delivery of up-to-date modules on comprehensive treatment and care of people living with HIV/AIDS can be contextualized and customized to some of the most-used mobile devices. Particular attention should be given to the adaptation of the educational material to the small screen size and to the performance of the program development in the different operating systems.

## Competing interests

The authors declare that they have no competing interests.

## Authors' contributions

MZ wrote the grant proposal, contributed to the educational content development, wrote reports and drafted the manuscript; DI participated as principal investigator, developed educational content and coordinated the project in Peru; CK participated to the project design and to the coordination and helped drafting the manuscript; JE participated to the project design and to the stakeholders involvement; LF, EL, IdW, WCL realized the software applications and participated into the project design; VS performed the statistical analysis; LL conceived the principal idea and looked for funding opportunities. All authors read and approved the final manuscript.

## Supplementary Material

Additional file 1 List of CME modules and learning objectives Click here for file
