# Supplementary material for: Mobile learning for HIV/AIDS healthcare worker training in resource-limited settings
Source: AIDS Res Ther. 2010 Sep 8;7:35. doi: 10.1186/1742-6405-7-35 (PMC2942790; doi:10.1186/1742-6405-7-35)
Supplement: Additional file 1 — List of CME modules and learning objectives [file 1742-6405-7-35-S1.DOC]

## Annex 1 - List of CME modules and learning objectives

| **MODULE** | **Learning objectives** |
| --- | --- |
| I. When to start antiretroviral therapy (HAART early start) | 1. Discuss the rationale for earlier versus later HAART initiation 2. Understand why it is better to start treatment when CD_4_ <350 compared to CD_4_ <200 |
| II. Diagnosis and treatment of lipodystrophy | 1. How to make the diagnosis of lipodystrophy 2. Which antiretrovirals are more associated with the development of lipodystrophy 3. What measures can prevent the development of lipodystrophy 4. What is the treatment for lipodystrophy |
| III. Tenofovir and Abacavir use | *Abacavir*   1. When to use Abacavir 2. Know how often hypersensitivity syndrome due to Abacavir occurs 3. How to diagnose the hypersensitivity syndrome due to Abacavir 4. What is the treatment for hypersensitivity syndrome   *Tenofovir*   1. When to use Tenofovir 2. What are the advantages to using Tenofovir compared to Zidovudine, Stavudine, Didanosine 3. Know major contraindications for the use of Tenofovir 4. Understand why the monitoring of renal function is necessary |
| IV. Virological failure and switch to second-line HAART | 1. Identify the criteria for virological failure 2. Know the main drug-resistance mutations after failing a first-line NNRTI-based HAART 3. Know the criteria for selecting the most appropriate second-line combination 4. Differences between Atazanavir/ritonavir versus Lopinavir/ritonavir second-line regimens |
| V. Use of protease inhibitors in pregnant women | 1. Use of Atazanavir/ritonavir and Lopinavir/ritonavir during pregnancy 2. Safety profile of Atazanavir/ritonavir vs. Lopinavir/ritonavir during pregnancy |
